# Superior efficacy of 100-Hz transcutaneous electrical nerve stimulation in reducing post-stroke spasticity: a systematic review and meta-analysis

**DOI:** 10.1186/s12984-025-01744-3

**Published:** 2025-10-09

**Authors:** Yingxiu Diao, Xiaomin Niu, Jiahao Huang, Chong You, Xiaoying Lin, Jiaxin Pan, Jianghua Cheng

**Affiliations:** 1 Department of Rehabilitation Medicine, South China Hospital, Medical School, Shenzhen University, Shenzhen, 518116 China; 2 School of Rehabilitation Medicine, Gannan Medical University, Ganzhou, 341000 Jiangxi China

**Keywords:** Stroke, Spasticity, Transcutaneous electrical nerve stimulation, TENS, Meta-analysis, Neurorehabilitation, High-frequency stimulation

## Abstract

**Background:**

Post-stroke spasticity is a prevalent complication of upper motor neuron injury, hindering motor recovery, independence, and quality of life. Transcutaneous electrical nerve stimulation (TENS) has been proposed as a non-invasive strategy to modulate neural excitability and reduce spasticity. However, its clinical efficacy remains uncertain due to heterogeneity in stimulation protocols and patient characteristics. This systematic review and meta-analysis aimed to assess the overall effectiveness of TENS in managing post-stroke spasticity and to examine potential differences in outcomes across stimulation frequencies and stroke phases.

**Methods:**

Databases searched included PubMed, Embase, Web of Science, Scopus, PEDro, and the Cochrane Library up to March 2025. The primary outcome was spasticity severity, assessed using the Modified Ashworth Scale (MAS) or Composite Spasticity Score (CSS). Subgroup analyses were conducted by stimulation frequency and stroke stage. Standardized mean differences (SMDs) were calculated using a random-effects model. Risk of bias was assessed using the Cochrane RoB 2.0 tool.

**Results:**

Seventeen randomized controlled trials (RCTs) involving 913 participants were included. TENS significantly reduced post-stroke spasticity compared to controls (SMD = − 0.64; 95% CI: − 0.91 to − 0.37; *P* < 0.001; I² = 69%). Subgroup analysis revealed the greatest effect in the acute phase (SMD = − 1.77), followed by subacute (SMD = − 0.61) and chronic phases (SMD = − 0.44) (p for subgroup difference < 0.001). TENS at 100 Hz yielded significant improvement (SMD = − 0.69), whereas lower frequencies (< 100 Hz) did not reach statistical significance. However, between-frequency group differences were not statistically significant (*P* = 0.67). Sensitivity analyses confirmed the robustness of the findings. Egger’s test suggested potential publication bias (*P* = 0.008).

**Conclusions:**

TENS is a safe and effective intervention for reducing post-stroke spasticity, especially when applied during the acute phase. High-frequency stimulation at 100 Hz may confer greater benefits, though further standardized studies are needed to validate optimal parameters and timing. These results support the early incorporation of 100 Hz TENS into comprehensive stroke rehabilitation protocols.

*PROSPERO registration number*: CRD 420251029133.

**Supplementary Information:**

The online version contains supplementary material available at 10.1186/s12984-025-01744-3.

## Introduction

Post-stroke spasticity is a prevalent and disabling manifestation of upper motor neuron syndrome, affecting approximately 17–43% of stroke survivors, particularly beyond the subacute phase [1, 2]. It imposes considerable functional limitations, compromises quality of life, and significantly increases healthcare costs and caregiver burden [3]. Effective management of spasticity is essential to facilitate motor recovery and optimize functional independence in activities of daily living (ADL). Clinically, spasticity is most frequently assessed using the Modified Ashworth Scale (MAS), which evaluates resistance to passive stretch. Although easy to administer, MAS has limitations in terms of inter-rater reliability and sensitivity to change, prompting researchers to complement it with other tools such as the Tardieu Scale or electromyographic analysis in some studies. Among various rehabilitative interventions, transcutaneous electrical nerve stimulation (TENS), a noninvasive, low-cost, and easily administrable modality, has garnered attention for its potential role in mitigating spasticity [4–6]. TENS is hypothesized to exert antispastic effects through several neurophysiological mechanisms, including presynaptic inhibition of α-motoneurons via Ia afferent modulation [7], enhancement of reciprocal inhibition [8], and long-term depression of spinal reflex excitability [9]. TENS is suggested to influence corticomotor plasticity via activation of descending inhibitory pathways and sensorimotor integration [10, 11]. These mechanisms are supported by both neurophysiological and neuroimaging studies showing altered excitability in the corticospinal and spinal circuits following sensory-level electrical stimulation [12].

Despite increasing research interest, the clinical effectiveness of TENS in treating post-stroke spasticity remains inconclusive. This is mainly due to the heterogeneity among stimulation parameters, treatment protocols and outcome measures (e.g. frequency, intensity pulse duration, electrode location) [4–6, 13–18]. Frequencies employed in clinical trials have ranged widely from low to high, with no consensus on optimal parameter settings. Additionally, electrode placement varies across studies, including application over muscle bellies, peripheral nerves, and acupoints, each potentially inducing distinct physiological effects. Despite its increasing application, the timing of TENS in relation to the acute, subacute, and chronic stages of stroke recovery has not been thoroughly examined. Since neuroplasticity varies significantly throughout the course of recovery, the effectiveness of TENS may depend on the specific stage during which it is delivered.

Previous systematic reviews have examined the general efficacy of TENS in motor rehabilitation after stroke [19, 20], but many have focused broadly on motor outcomes without specifically addressing spasticity as a primary endpoint. Furthermore, few have performed subgroup analyses based on stroke phase or stimulation frequency, limiting the clinical applicability of their findings. To our knowledge, no meta-analysis has comprehensively compared the effects of different TENS frequencies across the various stroke phases while also conducting robust subgroup and sensitivity analyses. To overcome these limitations, we performed a systematic review and meta-analysis of randomized controlled trials (RCTs) evaluating the effectiveness of TENS in reducing post-stroke spasticity. Specifically, this review aims to (1) compare the therapeutic effects of different TENS frequencies; (2) examine the influence of stroke phase (acute, subacute, chronic) on clinical outcomes; and (3) conduct subgroup and sensitivity analyses to identify parameter- and phase-specific therapeutic effects. This study aims to provide more clinically relevant and targeted recommendations on the use of TENS for post-stroke rehabilitation by combining evidence from multiple variables.

## Methods

### Protocol and registration

The Cochrane Handbook for Systematic Reviews of Interventions [21] and PRISMA guidelines were used to conduct this systematic review [22]. The protocol was prospectively registered on the International Prospective Register of Systematic Reviews (PROSPERO; registration number: CRD420251029133).

### Information sources and search

Six major databases were systematically searched: MEDLINE via PubMed, Embase, the Cochrane Library, Web of Science, Scopus, and the Physiotherapy Evidence Database (PEDro). This search included all published material from the database’s inception until March 15, 2025. Medical Subject Headings and Free-Text Terms were used to maximize retrieval accuracy. Core search terms included “transcutaneous electrical nerve stimulation,” “TENS,” “transcutaneous neuromuscular electrical stimulation,” “spasticity,” and “stroke.” Boolean operators (AND, OR) were used to structure search strings according to the indexing protocols of each database. In addition to electronic searches, reference lists of all included studies and relevant systematic reviews or meta-analyses were manually screened to identify potentially eligible trials not captured through database queries. Study selection was performed in two stages: an initial screening of titles and abstracts, followed by full-text review based on predefined inclusion and exclusion criteria. The study selection process was carried out independently by two reviewers (K.D. and C.Y.). The reviewers worked independently during both screening phases, and any discrepancies were resolved through discussion. If consensus could not be reached, a third reviewer (J.C.) was consulted for adjudication. The full search strategies, including database-specific syntax and applied filters, are detailed in the Supplemental Material.

### Eligibility criteria

The PICOS framework guided the selection of studies based on eligibility criteria. The RCTs were eligible if they included adult participants (18 years and older) who had a diagnosis of stroke, either ischemic or haemorrhagic with documented spasticity in at least one extremity. All phases of stroke recovery—acute, subacute, and chronic—were considered. The intervention of interest was TENS, applied either as a standalone treatment or in combination with conventional rehabilitation. TENS protocols could include stimulation over peripheral nerves, muscle bellies, or acupuncture points. Eligible comparators included sham TENS, placebo stimulation, usual care, or other forms of conventional therapy. Studies were required to include at least one primary quantitative outcome of spasticity, measured using clinically validated tools, such as the Modified Ashworth Scale (MAS), Tardieu Scale or Composite Spasticity Score (CSS). Secondary outcomes, such as motor functions (e.g., Fugl-Meyer Assessment [FMA]) and activities of daily living (e.g., Barthel Index [BI]) were also considered when reported.

Studies were excluded if they (1) were not randomized controlled trials (e.g., case reports, observational studies, reviews); (2) included participants with non-stroke-related spasticity or unclear diagnosis; (3) lacked quantifiable spasticity outcomes; (4) used interventions not consistent with conventional TENS (e.g., invasive stimulation, implanted devices); or (5) were not published in English.

### Data extraction

Data extraction was performed independently by two reviewers (Y.D. and J.P.) using a standardized and piloted extraction form. Prior to the formal extraction process, the form was trialed on a sample of eligible studies to ensure clarity and consistency. Disagreements between reviewers were resolved through discussion or adjudication by a third reviewer (J.C.). The following information was extracted from each included study: (1) bibliographic details (first author, year of publication, country); (2) participant characteristics (sample size, mean age, sex distribution, stroke type and phase, affected limb); (3) intervention details (TENS frequency, intensity, pulse width, duration per session, total number of sessions, electrode placement, and whether TENS was applied alone or in combination with other therapies); (4) comparator characteristics; (5) outcome measures and assessment time points; and (6) key findings, including pre- and post-intervention data for all relevant outcomes. When data were reported in figures or incomplete formats, digital extraction tools (e.g., WebPlotDigitizer) were used, and study authors were contacted via email when necessary to obtain missing or clarifying information. If standard deviations were not reported, they were calculated from standard errors, confidence intervals, or interquartile ranges, following the Cochrane Handbook guidelines. All extracted data were cross-verified by a second reviewer to minimize transcription errors and ensure accuracy. Any inconsistencies were addressed through consensus. The final dataset was used for quantitative synthesis in the meta-analysis.

### Risk of bias assessment

Two reviewers (J.H. and X.L.) will independently apply the Cochrane Risk of Bias tool 2.0 to assess the methodological quality of included studies [21]. This tool evaluates five key domains: The randomization process will be evaluated for deviations from intended interventions, incomplete outcome data, the measurement of outcome and the selection of reported results. Each domain is rated either as low, moderate, or high in terms of the risk of bias. Discussions between the reviewers resolved disagreements or, if necessary, consultation with a third reviewer. In the case of studies that did not provide enough information for a clear judgement, we contacted authors to get clarification. The risk-of-bias judgments were used to inform the interpretation of results, and sensitivity analyses were conducted to explore the robustness of findings based on study quality.

### Statistical analysis

To account for the variability of outcome measurement methods across studies, we calculated standardized mean difference (SMD) and 95% confidence intervals. If multiple outcomes were reported, we prioritized the scale that was most widely used, validated, and standardized (e.g. MAS of spasticity). We used established conversion formulas to estimate means and standard deviations from medians and interquartile ranges where necessary. The Chi-square (Cochran’s Q) test was used to assess heterogeneity between studies. This data is quantified using the I² statistics. A p-value below 0.10 or an I² above 50% was considered to indicate substantial heterogeneity. In this case, a random effects model was used (DerSimonian-Laird method). A fixed effects model would be used if not. For each study included, forest plots were created to display visually the effect sizes as well as confidence intervals. Subgroup analyses were performed to explore heterogeneity based on stroke phases (acute or subacute) and stimulation frequencies. To assess robustness, sensitivity analyses were conducted by sequentially eliminating individual studies. When at least 10 publications were available on a particular outcome, publication bias could be assessed. Egger’s regression and visual inspection of funnel plots were used to detect effects from small studies. Trim-and-fill was used to assess the impact of publication bias on pooled effects in cases where it was suspected.

## Results

### Study selection and characteristics

**Fig. 1:**
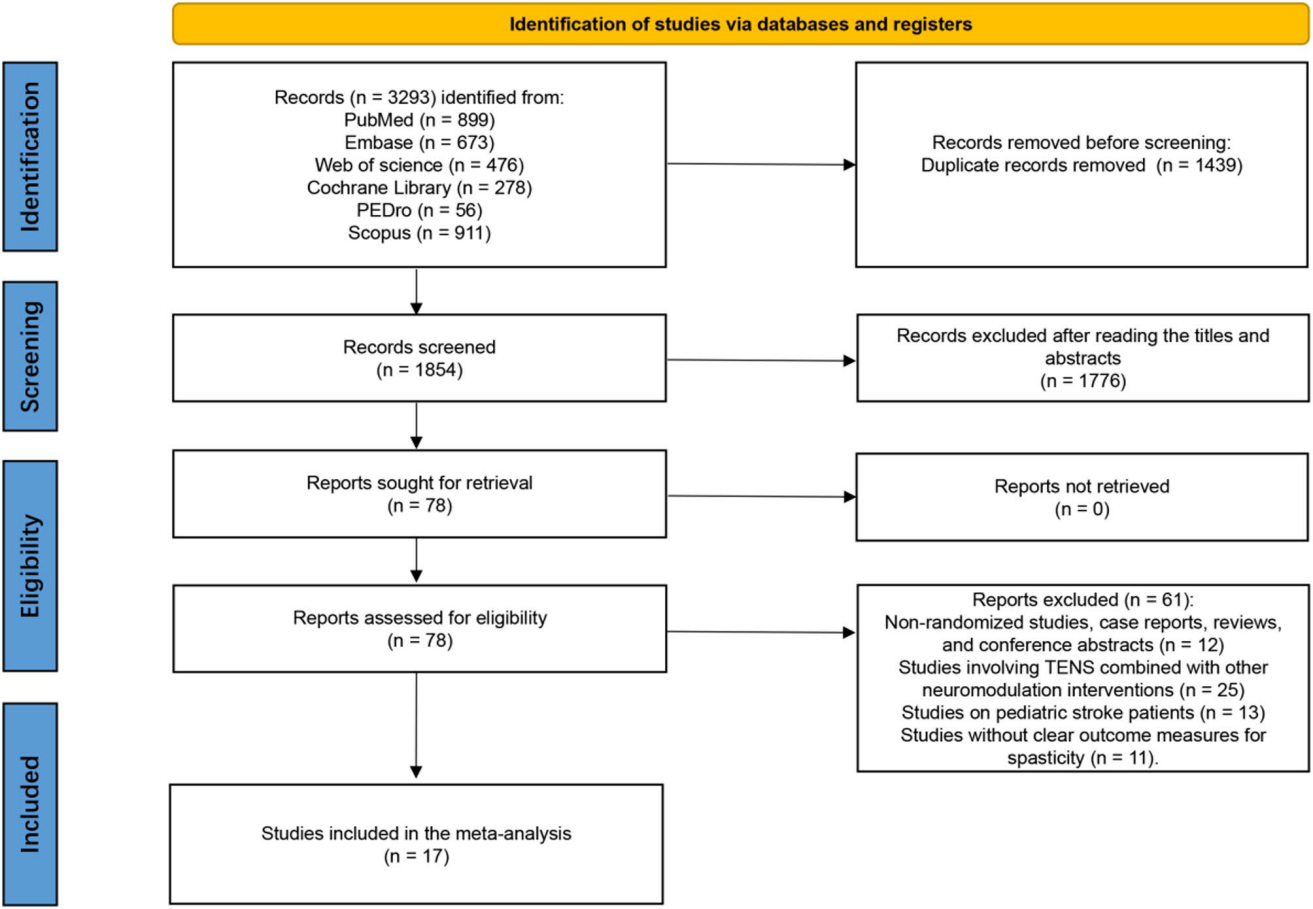
PRISMA flow diagram of the literature search and study selection process

The study selection process is summarized in Fig. 1. A total of 3,293 records were identified through electronic database searches, including 899 from PubMed, 673 from Embase, 476 from Web of Science, 278 from the Cochrane Library, 56 from PEDro, and 911 from Scopus. After removing 1,439 duplicates, 1,854 unique articles remained for title and abstract screening. Of these, 78 articles were deemed potentially eligible and were retrieved for full-text review. Following full-text assessment, 61 articles were excluded for various reasons, and 17 RCTs [23–39] met the inclusion criteria and were included in the final meta-analysis.

All studies adopted RCT design and were published between 1998 and 2025. China accounted for the largest number of studies (*n* = 8) [24, 28, 29, 32, 36–39]. Korea contributed four studies [25, 30, 31, 33], followed by Turkey [35], Italy [23], the Netherlands [26], India [27], and Sweden [34], each contributing one. Stroke phase at the time of intervention varied among studies. One study [38] exclusively included patients in the acute phase, while Wang [36] enrolled patients spanning acute, subacute, and chronic stages. Five trials were conducted in the subacute phase [26, 27, 29, 30, 39], in addition to the mixed-phase study by Wang [36]. Chronic stroke survivors were targeted in eight studies [23–25, 28, 31–34], again supplemented by the mixed-phase study. Two studies [35, 37] did not report the duration since stroke onset. Control interventions varied across the included trials. Eleven studies employed sham or placebo TENS as the control condition, enabling blinding of participants and assessors. Three studies used conventional rehabilitation as the comparator, while another three compared TENS combined with traditional therapies (e.g., Bobath, task-related training, stretching) versus those therapies alone. The remaining studies used either no treatment or standard care. Electrode placement strategies were heterogeneous. Several studies targeted specific muscle groups such as the triceps brachii, gastrocnemius, quadriceps, extensor digitorum communis, deltoid, infraspinatus, teres minor, and supraspinatus muscles. Others applied stimulation to peripheral nerves, particularly the peroneal nerve. A subset of studies used acupuncture points as electrode sites [27–29, 32, 36–38], most commonly ST36, LV3, GB34, UB60, LI10, TE5, LI15, LI14, LI11, SJ5, and LI4. Some studies combined multiple anatomical targets within the same protocol. Stimulation parameters varied but followed consistent patterns. Most studies applied current intensity at a “bearable level” or set the output to two to three times the sensory threshold. Frequency was predominantly 100 Hz, reported in 12 studies [25, 27–33, 35, 37–39], though other frequencies ranged from 1.7 Hz to 100 Hz. Pulse width was reported in 10 studies [24, 25, 27–33, 38], with 200 microseconds being the most common setting, followed by 300 microseconds in two studies. Treatment protocols also demonstrated variability. Duration per session ranged from 20 to 60 min, with the most common regimen being 5 sessions per week. The total intervention course ranged from 1 to 8 weeks across trials, with one study [34] extending to 3 months. These characteristics highlight substantial diversity in methodological approaches, reinforcing the importance of subgroup and sensitivity analyses in subsequent quantitative synthesis. A detailed summary of intervention parameters and study features is provided in Table 1.

**Table 1:** Characteristics of included randomized controlled trials

Author, (Year)	Country	Study Design	Intervention	Onset Time	Sample Size (M/F)	Electrode Placement	Current Intensity	Frequency (Hz)	Pulse Width	Treatment time
Tekeoğlu 1998	Turkey	RCT	E: TENS C: placebo	NR	E: 30 (17/13) C: 30 (14/16)	Musculus triceps brachii	Bearable level	100	NR	30 min/session, 5 sessions/w, 8 w
Baricich 2008	Italy	RCT	E: TENS and stretching C: stretching	Chronic	E: 8 (5/3) C: 7 (5/2)	Gastrocnemius	Bearable level	5	NR	60 min/session, 5 sessions/w, 1 w
Chen 2005	China	RCT	E: TENS C: Sham TENS	Chronic	E: 12 (7/5) C: 12 (7/5)	Gastrocnemius	Constant-current	20	200	20 min/session, 6 sessions/w, 4 w
Cho 2013	Korea	RCT	E: TENS C: placebo	Chronic	E: 22 (14/8) C: 20 (13/7)	Gastrocnemius	2 to 3 times the sensory threshold	100	200	60 min/session, 1 session
de Jong 2013	Netherlands	RCT	E: TENS and stretching C: Sham TENS and stretching	Subacute	E: 23 (15/8) C: 23 (12/11)	extensor digitorum communis muscle/deltoid muscle/infraspinatus/teres minor muscle	Bearable level	35	300	45 min/session, 5 sessions/w, 8 w
Deshmukh 2013	India	RCT	E: TENS and TRT C: TRT	Subacute	E: 15 C: 15	St 36, Lv 3, GB 34, and Bl 60	Bearable level	100	200	60 min/session, 5 sessions/w, 5 w
Wang 2023	China	RCT	E: TENS C: Sham TENS	Acute(*n* = 72), subacute(*n* = 92) and Chronic(*n* = 40)	E: 102 (77/25) C: 102 (72/30)	LI10 and TE5	Maximum tolerable	2	300	30 min/session, 5 sessions/w, 6 w
Hui-Chan 2009	China	RCT	E_1_: TENS and TRT C_1_: placebo and TRT E_2_: TENS C_2_: no treatment	Chronic	E_1_: 27 C_1_: 25 E_2_: 28 C_2_: 29	St 36, Lv 3, GB 34, and UB 60	Two to three times the sensory threshold	100	200	60 min/session, 5 sessions/w, 4 w
Jung 2017	Korea	RCT	E: TENS C: Sham TENS	Subacute	E: 20 (11/9) C: 20 (12/8)	Peroneal nerve	Two times the sensory threshold	100	200	60 min/session, 5 sessions/w, 6 w
Park 2014	Korea	RCT	E: TENS C: Sham TENS	Chronic	E: 15 (12/3) C: 14 (8/6)	Quadriceps and gastrocnemius	90% amplitude using the sub-sensory threshold	100	200	30 min/session, 5 sessions/w, 6 w
Kim 2013	Korea	RCT	E: TENS and TRT C: placebo and TRT	Chronic	E: 15 (9/6) C: 15 (8/7)	Muscle belly of triceps and wrist extensors	Two to three times the sensory threshold	100	200	30 min/session, 5 sessions/w, 4 w
Ng 2007	China	RCT	E_1_: TENS and TRT C_1_: placebo and TRT E_2_: TENS C_2_: no treatment	Chronic	E_1_: 21 (16/5) C_1_: 20 (17/3) E_2_: 19 (17/2) C_2_: 20 (17/3)	St 36, Lv 3, GB 34, and UB 60	Two to three times the sensory threshold	100	200	60 min/session, 5 sessions/w, 4 w
Sonde 2000	Sweden	RCT	E: TENS C: placebo	Chronic	E: 18 (14/4) C: 10 (6/4)	NR	NR	1.7	NR	3 m
Wang 2025	China	RCT	E: TENS C: Sham TENS	NR	E: 20 (14/6) C: 20 (16/4)	LI15, LI14, LI11, LI10, SJ5 and LI4	Bearable level	100	NR	30 min/session, 3 sessions/w, 6 w
Hussain 2013	China	RCT	E: TENS and Bobath inhibitory C: Bobath inhibitory	Subacute	E: 15 (8/7) C: 15 (10/5)	St 36, Lv 3, GB 34, and UB 60	Two to three times the sensory threshold	100	200	30 min/session, 5 sessions/w, 4 w
Yan 2009	China	RCT	E: TENS C_1_: placebo C_2_: standard rehabilitation	Acute	E: 19 (9/10) C_1_: 19 (10/9) C_2_: 18 (9/9)	St 36, Lv 3, GB 34, and Bl 60	Bearable level	100	200	60 min/session, 5 sessions/w, 3 w
Zhou 2018	China	RCT	E: TENS and conventional rehabilitation C: conventional rehabilitation	Subacute	E: 32 (26/6) C: 18 (15/3)	Supraspinatus	Produce visible contractions of shoulder abductors without any discomfort	100	100	60 min/session, 5 sessions/w, 4 w

* E, experimental group; C, control group; RCT, randomized controlled trail; TENS, transcutaneous electrical nerve stimulation; TRT, task-related training; CSS, composite spasticity score; NR, not reported

### Risk of bias assessment

**Fig. 2:**
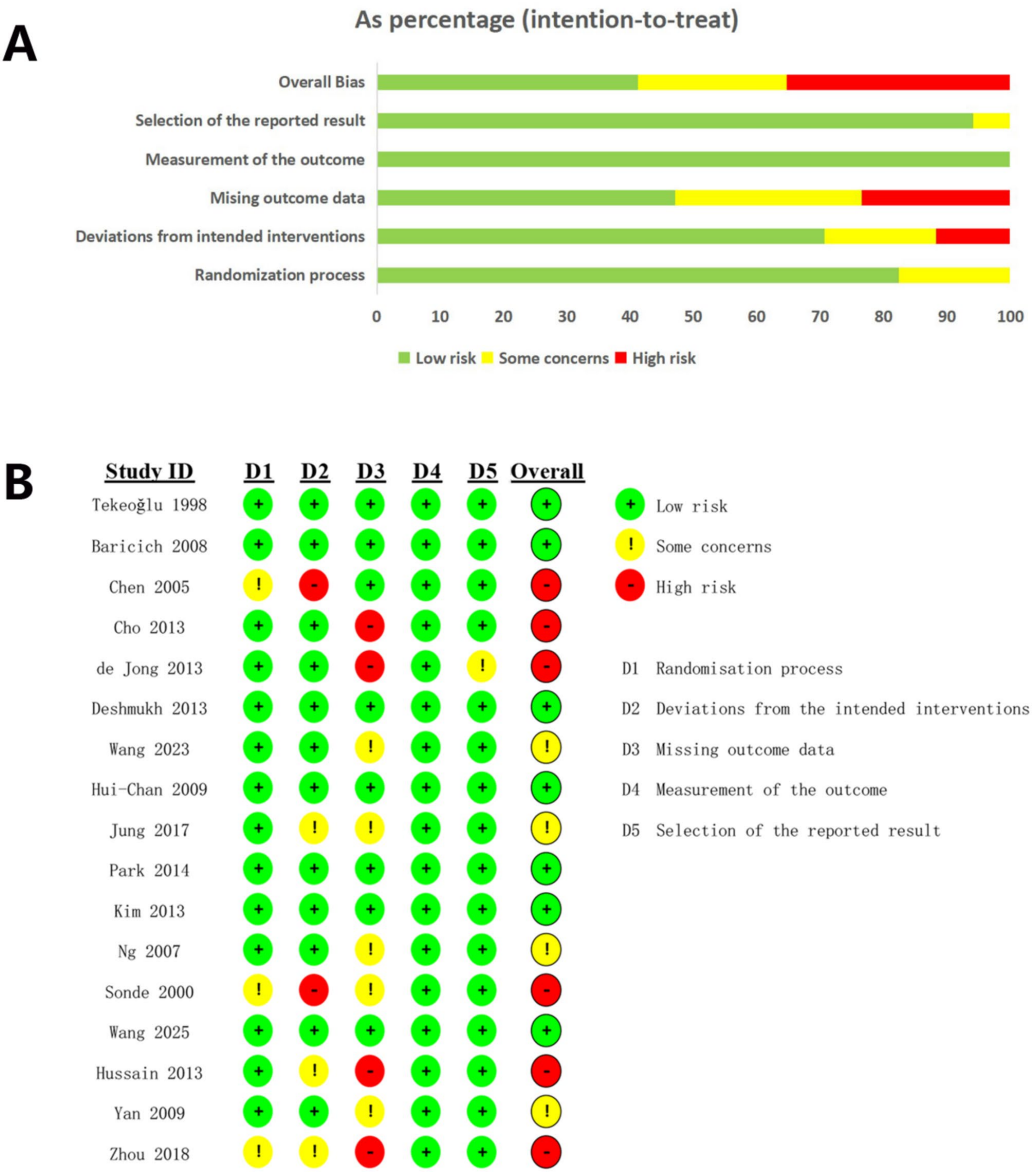
Risk of bias assessment of the included studies using the Cochrane Risk of Bias 2.0 tool. (A) Summary of risk of bias across all studies by domain, presented as percentages. (B) Domain-specific risk of bias for each individual study

The risk of bias in the included randomized controlled trials was evaluated using the RoB 2 tool, with results visually summarized in Fig. 2. Overall, the methodological quality of the studies was acceptable. Regarding randomization, three studies [24, 31, 39] lacked sufficient detail on their sequence generation methods and were thus rated as having ‘some concerns’. In contrast, the remaining studies clearly described the use of random number tables or computer-generated sequences and were classified as ‘low risk’. For deviations from intended interventions, two studies [24, 39] were classified as “high risk” due to substantial deviations from the planned protocol without adequate justification, and three others [31, 32, 36] were assessed as having “some concerns.” Regarding missing outcome data, four studies [24, 29, 32, 39] showed considerable attrition or inadequate reporting, resulting in a “high risk” judgment, whereas five studies [23, 26, 28, 31, 38] were rated as having “some concerns” due to moderate levels of missing data or unclear handling procedures. In contrast, nearly all studies demonstrated low risk in the domains of outcome measurement and selective reporting, with only one study [39] receiving a rating of “some concerns” in both domains due to insufficient reporting clarity. Taken together, although several trials exhibited risks in one or more domains, particularly concerning intervention fidelity and data completeness, the majority of the included studies adhered to rigorous methodological standards. These findings underscore the methodological variability among included trials and highlight the importance of interpreting pooled results within the context of individual study quality.

### Primary outcome

#### Spasticity

**Fig. 3:**
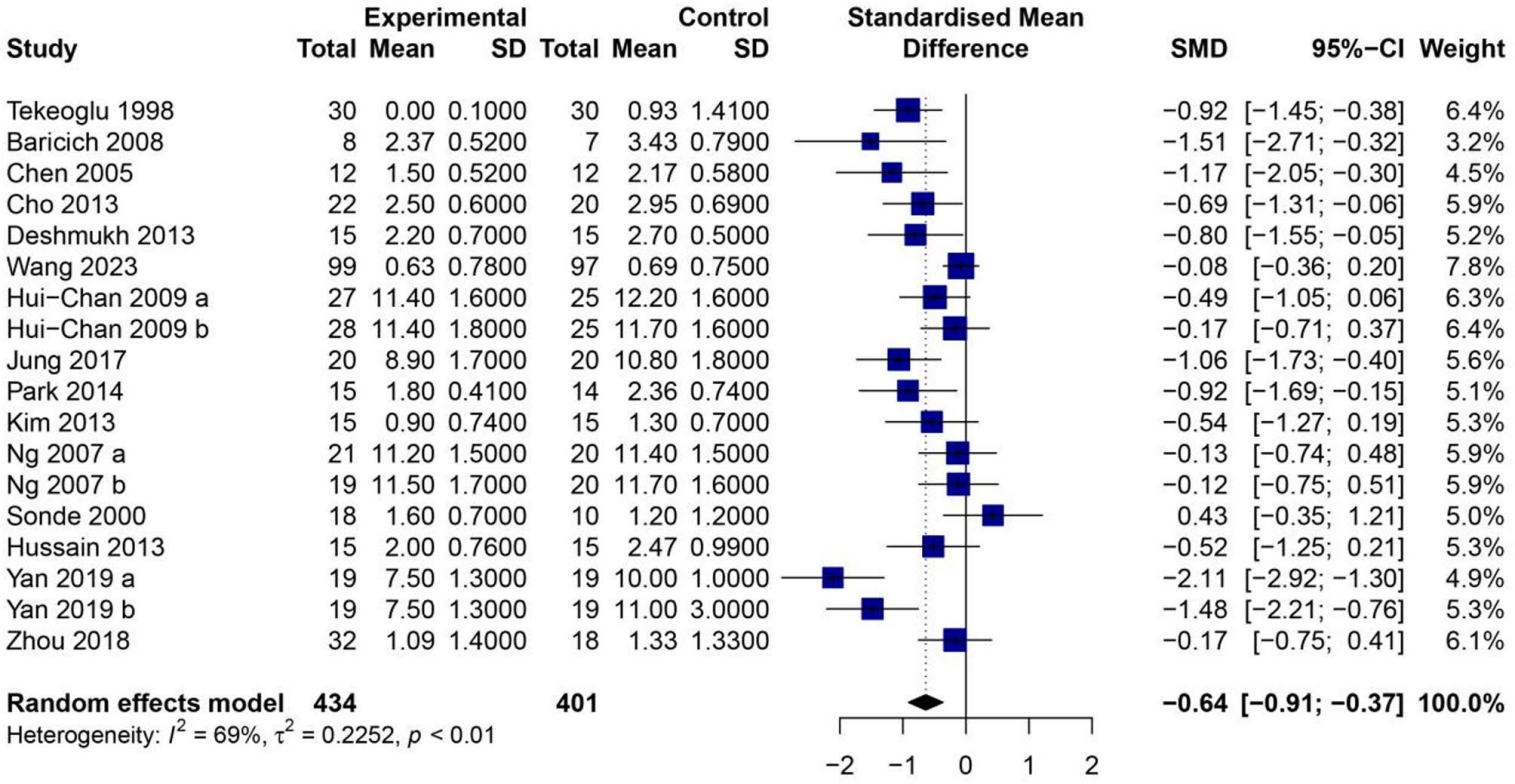
Forest plot of TENS on spasticity score for stroke patients

The included studies assessed post-stroke spasticity using two different outcome measures: the MAS and the CSS. Specifically, 13 studies employed the MAS, while four studies used the CSS. Given the variation in measurement instruments, SMD was used to synthesize the effect sizes across trials. The heterogeneity test revealed significant variability among the included studies (*I*² = 69%, *P* < 0.01), indicating moderate to high heterogeneity. Consequently, a random-effects model was applied to account for between-study differences. As illustrated in Fig. 3, the meta-analysis demonstrated that TENS significantly reduced spasticity scores compared to control interventions. The pooled SMD was − 0.64 (95% CI: − 0.91 to − 0.37, *P* < 0.01), suggesting a moderate treatment effect in favor of TENS. This difference was statistically significant and indicates that TENS may effectively alleviate muscle hypertonia in stroke survivors.

**Fig. 4:**
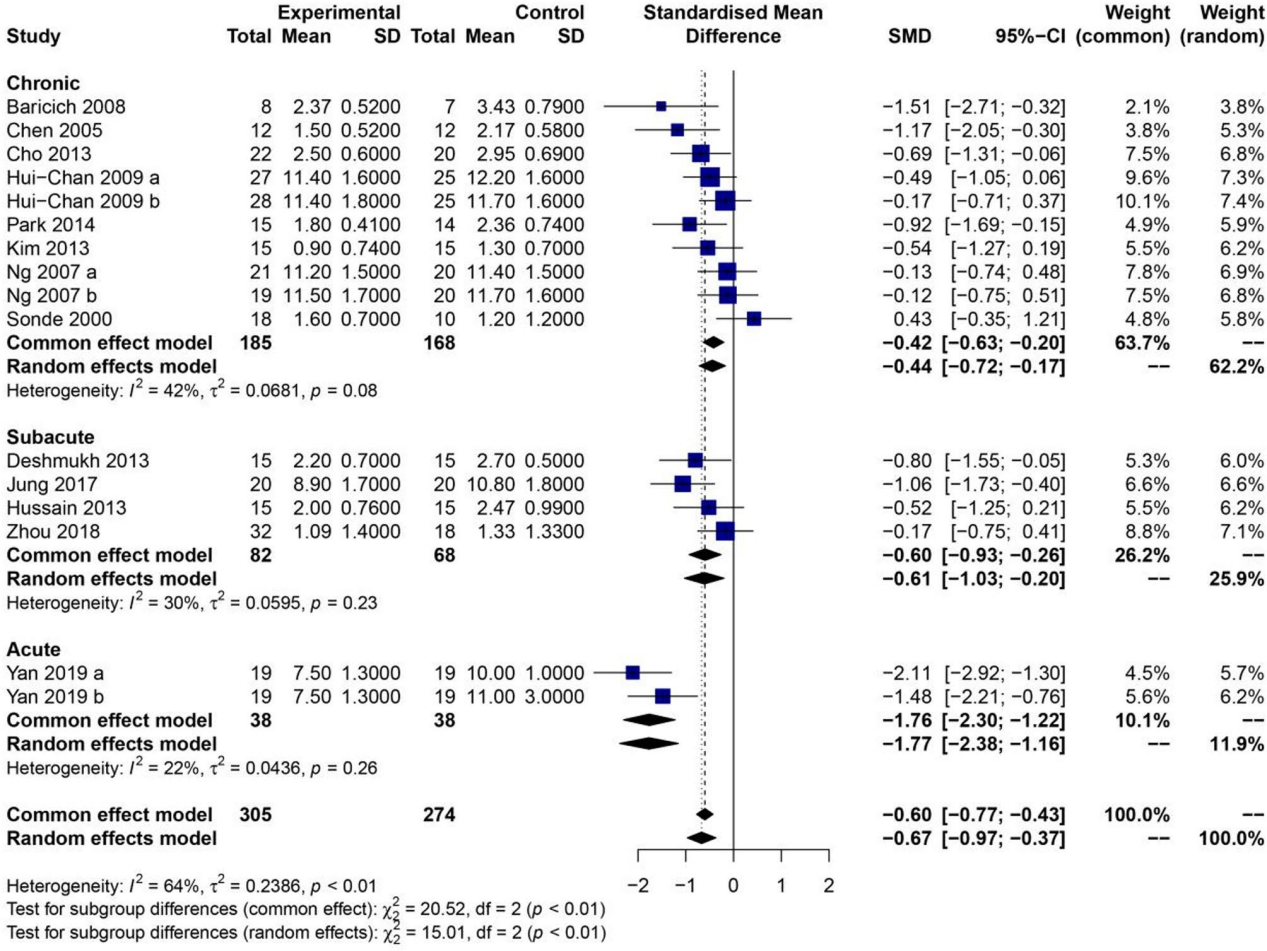
Forest plot of the effect of transcutaneous electrical nerve stimulation on spasticity scores in stroke patients, stratified by stroke chronicity

**Fig. 5:**
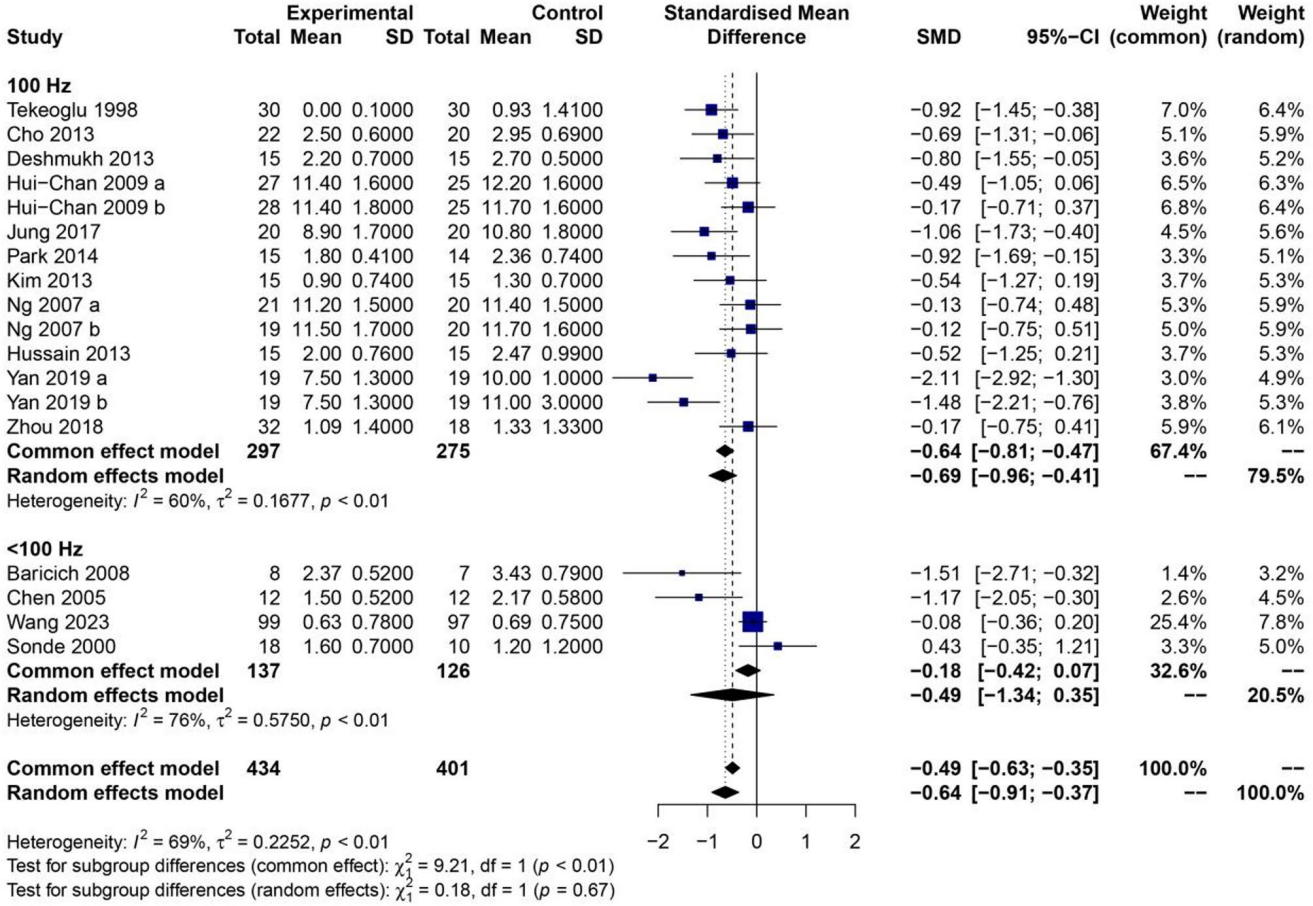
Forest plot of the effect of TENS on spasticity scores in stroke patients, stratified by stimulation frequency

To further explore potential sources of heterogeneity, subgroup analyses were performed based on stroke chronicity (Fig. 4) and stimulation frequency (Fig. 5). In the subgroup analysis stratified by stroke chronicity, the results showed that TENS significantly reduced spasticity scores in patients at all stages of recovery. Specifically, beneficial effects were observed in acute, subacute, and chronic stroke populations (all *P* < 0.01), indicating that the timing of intervention post-stroke does not substantially alter the therapeutic efficacy of TENS. Subgroup analysis based on stimulation frequency revealed more nuanced results. When the TENS frequency was set at 100 Hz, the experimental group exhibited a significantly greater reduction in spasticity scores compared to the control group (*P* < 0.01), with a statistically significant between-group difference. In contrast, when the stimulation frequency was below 100 Hz, no significant difference was observed between the TENS and control groups (*P* = 0.67). These findings suggest that higher-frequency stimulation may be more effective in modulating neuromuscular activity and reducing spasticity in stroke patients. Overall, the results of the meta-analysis support the use of TENS as an effective adjunct therapy for reducing spasticity after stroke, particularly when applied at higher frequencies. However, the observed heterogeneity and variation in stimulation parameters underscore the importance of further standardized, high-quality trials to confirm these findings and refine optimal treatment protocols.

### Secondary outcome

#### Motor function

**Fig. 6:**
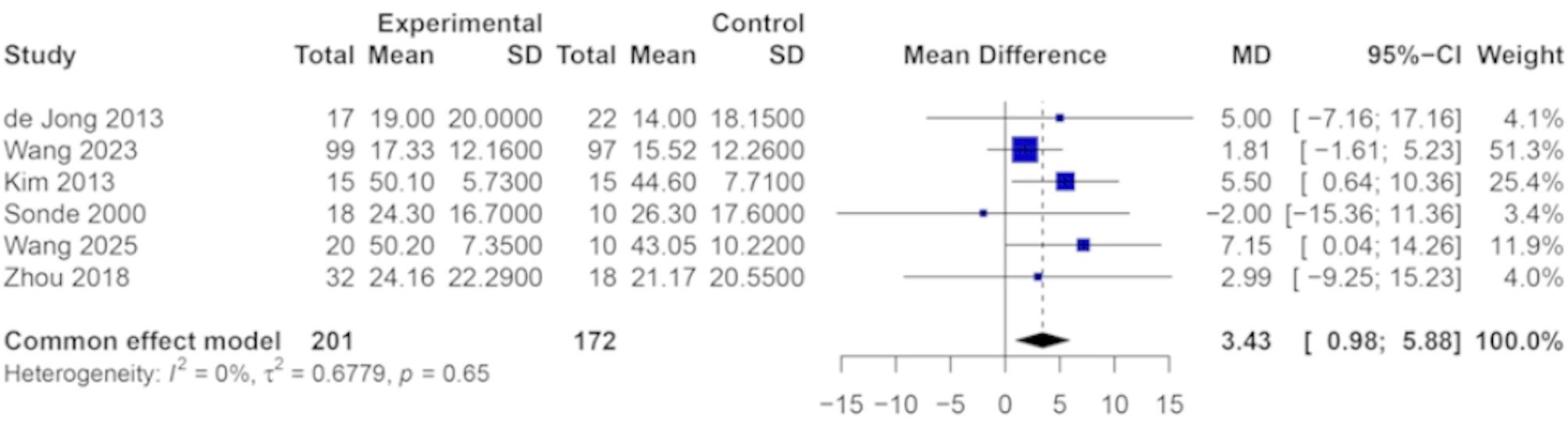
Forest plot of the effect of TENS on motor function for stroke patients

Six studies evaluated upper limb motor function using the FMA-UE as the outcome measure. Given the consistency of the measurement scale across all studies, mean difference (MD) was used to summarize the treatment effect. Heterogeneity analysis revealed minimal variability among studies (I² = 0%, *P* = 0.65), indicating a high level of consistency in treatment effects. Accordingly, a fixed-effects model was applied. As shown in Fig. 6, the pooled analysis demonstrated a statistically significant improvement in upper limb motor function in the TENS group compared to the control group. The combined MD was 3.43 (95% CI: 0.98 to 5.88, *P* < 0.01), suggesting a favorable effect of TENS on post-stroke motor recovery. Although the magnitude of improvement varied slightly across studies, the direction of effect consistently favored the experimental group.

### Activities of daily living

**Fig. 7:**
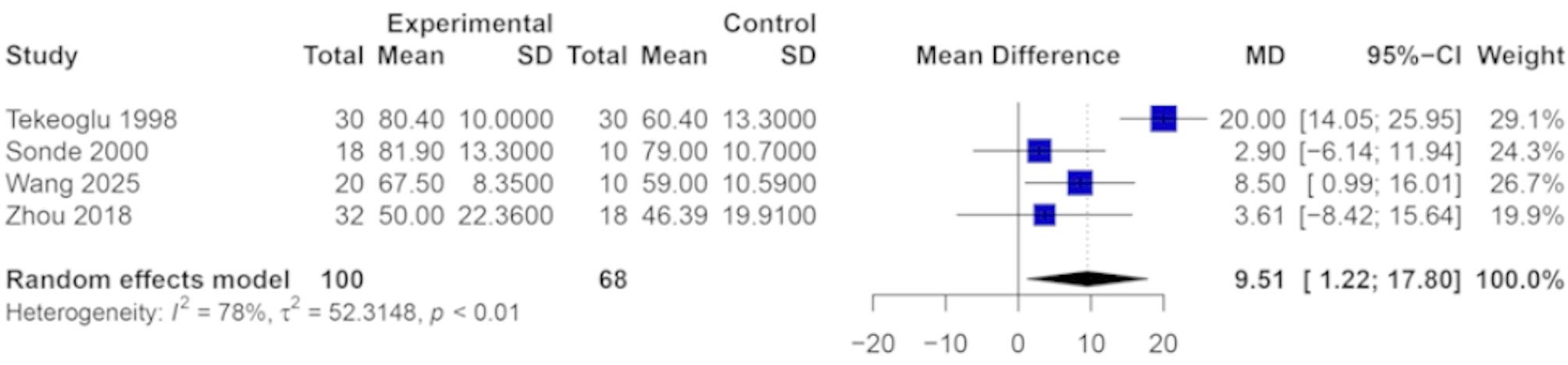
Forest plot of the effect of TENS on ADL for stroke patients

Four studies evaluated the impact of TENS on ADL using the BI as the primary outcome measure. Due to the uniformity of the measurement scale across studies, MD was used to estimate the overall treatment effect. However, heterogeneity analysis revealed substantial variability among the included trials (I² = 78%, *P* < 0.01), likely reflecting differences in study populations, intervention protocols, or timing of outcome assessment. Consequently, a random-effects model was employed to account for this inter-study heterogeneity. As illustrated in Fig. 7, the meta-analysis demonstrated a statistically significant improvement in ADL in favor of the TENS group. The pooled MD was 9.51 (95% CI: 1.22 to 17.80, *P* = 0.02), indicating that TENS may enhance functional independence in stroke survivors by improving their ability to perform daily activities.

### Sensitivity analysis

**Fig. 8:**
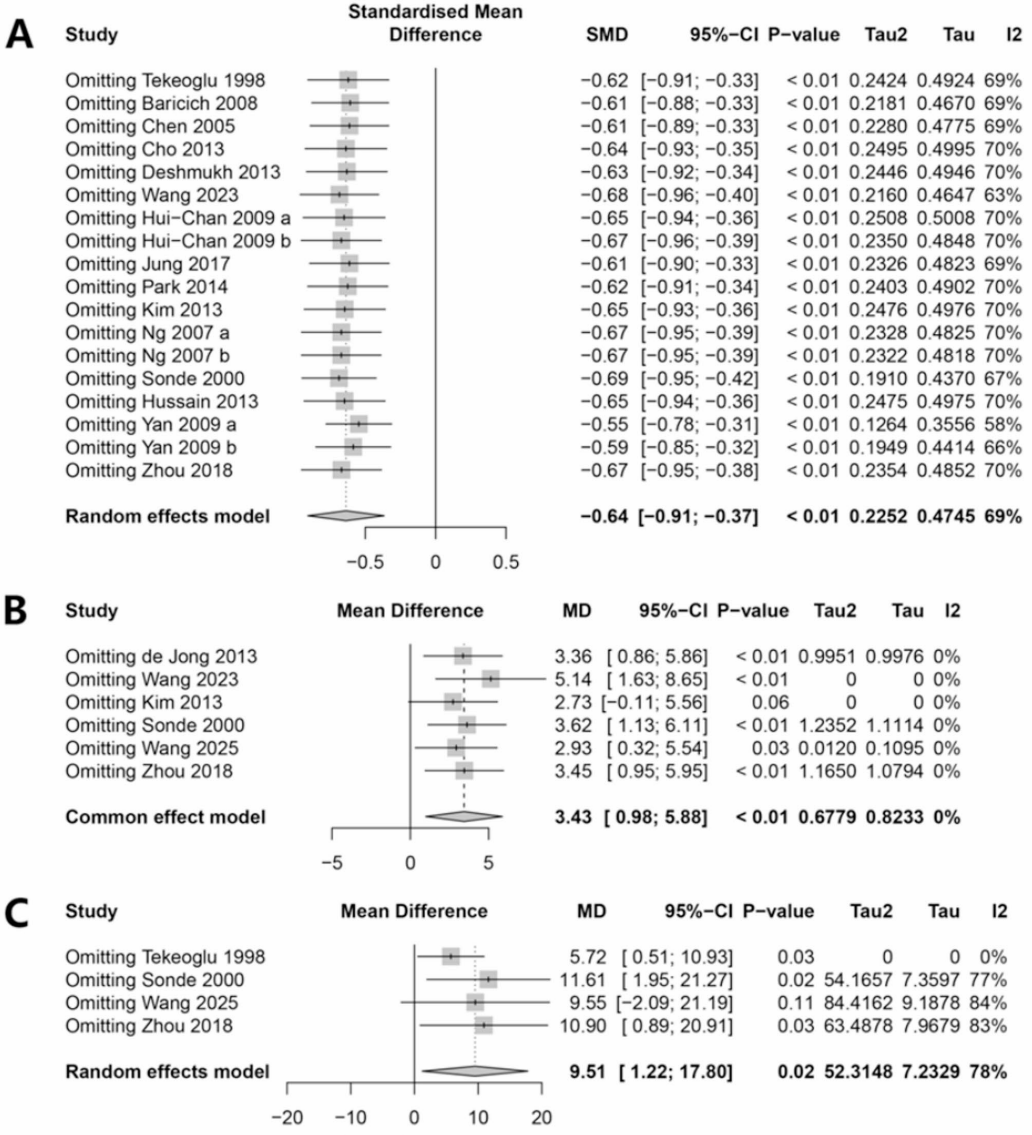
Plots of sensitivity analysis. A, spasticity score; B, motor function; C, activities of daily living

A leave-one-out sensitivity analysis was conducted to assess the robustness of the pooled estimates across the three primary outcomes (Fig. 8). For spasticity scores (Fig. 8A), the overall effect size remained stable regardless of which study was excluded, indicating a high degree of result reliability. In contrast, the pooled outcomes for motor function (Fig. 8B) and activities of daily living (ADL; Fig. 8C) were more sensitive to the exclusion of individual studies. Specifically, the magnitude and statistical significance of these results varied depending on the study removed. This sensitivity is likely attributable to the relatively small number of included studies (*n* = 6 for motor function; *n* = 4 for ADL), and, in the case of ADL, the presence of substantial heterogeneity (I² = 78%). These findings suggest that while the evidence supporting the effect of TENS on spasticity is robust, conclusions regarding its impact on motor function and ADL should be interpreted with caution.

### Publication bias

**Fig. 9:**
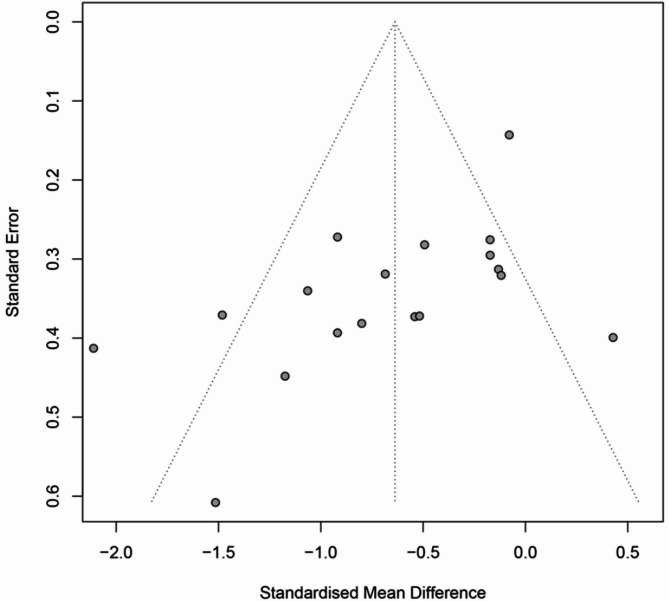
Funnel plot of publication bias for spasticity score

Among the included studies, only those reporting spasticity outcomes met the minimum threshold required for publication bias assessment. As shown in Fig. 9, the funnel plot appears visually symmetrical, suggesting no overt publication bias based on graphical inspection. However, Egger’s regression test revealed significant asymmetry (*P* = 0.008), indicating the potential presence of publication bias. This discrepancy underscores the limitations of relying solely on visual evaluation and highlights the need for statistical testing to detect small-study effects. Given this finding, the pooled effect estimate for spasticity should be interpreted with caution, as it may be influenced by selective reporting or underrepresentation of negative or null results.

## Discussion

This comprehensive systematic review and meta-analysis provides compelling evidence supporting the therapeutic efficacy of TENS in mitigating post-stroke spasticity and enhancing motor recovery. In particular, stimulation at 100 Hz demonstrated superior outcomes, with consistent improvements observed across spasticity severity, upper limb motor function, and ADL. These findings align with and extend previous literature, underscoring the relevance of stimulation parameters and timing in optimizing neuromodulatory outcomes.

The pooled effect size for spasticity reduction (SMD = − 0.64, 95% CI: − 0.91 to − 0.37, *P* < 0.001) indicates a moderate but clinically meaningful benefit of TENS over control interventions. This reinforces prior studies suggesting that TENS modulates spinal excitability and reduces hyperreflexia via peripheral afferent activation [24, 25, 28]. Importantly, subgroup analysis revealed that the therapeutic benefits were consistent across all stroke phases (acute, subacute, and chronic), suggesting that TENS may remain effective irrespective of the time since stroke onset. The superiority of 100 Hz stimulation over lower frequencies is particularly noteworthy. Studies such as Cho et al. [25] and Hui-Chan et al. [28] demonstrated that high-frequency TENS delivered at 2–3 times the sensory threshold significantly suppressed spasticity and improved dynamic balance. In contrast, studies using lower frequencies, such as 2–20 Hz, often reported non-significant outcomes or smaller effect sizes [23, 24, 35]. This frequency-dependent effect may be attributed to differential activation of supraspinal inhibitory circuits and more robust engagement of Aβ afferents [32, 34].

Beyond spasticity modulation, TENS was associated with improved upper limb motor function, as evidenced by a significant mean difference in FMA-UE scores (MD = 3.43, 95% CI: 0.98 to 5.88, *P* = 0.006). These improvements are consistent with mechanistic hypotheses suggesting that TENS enhances corticomotor excitability and facilitates neuroplasticity through increased afferent input [31, 37, 39]. The direction of effect was uniform across all six included studies, indicating that the intervention reliably benefits voluntary motor control. Similarly, TENS significantly enhanced ADL performance, with a pooled MD of 9.51 points on the Barthel Index (95% CI: 1.22 to 17.80, *p* = 0.02). Given that ADL recovery is a key rehabilitation goal, these findings highlight the functional relevance of electrical stimulation in post-stroke care. However, the high heterogeneity observed in the ADL-related outcomes (*I*² = 78%) suggests variability in treatment protocols, patient characteristics, or assessment timing, necessitating cautious interpretation. This study provides novel insights into the temporal applicability of TENS. Previous literature often focused on chronic stroke populations [28, 30, 31], whereas our analysis included patients across the spectrum of recovery. Subgroup findings demonstrated that TENS significantly reduced spasticity in acute, subacute, and chronic phases, suggesting that neuroplastic responsiveness to afferent input may be preserved across recovery stages. These findings support the early integration of TENS in stroke rehabilitation protocols, potentially capitalizing on the heightened neuroplasticity observed in the subacute phase [36].

The findings of our meta-analysis align with and extend previous reviews assessing the effects of TENS in stroke rehabilitation. Kwong et al. (2017) demonstrated that TENS significantly improved walking capacity and reduced lower limb spasticity [40]; however, their analysis was limited to lower extremity outcomes and did not include motor function or ADL measures. Our study expands on this by incorporating upper limb motor recovery and functional independence (BI), providing a more comprehensive evaluation. Mahmood et al. (2019) similarly found that TENS reduced lower limb spasticity when combined with physical therapy, particularly at higher frequencies and longer durations [20]. Our results support this, showing that 100 Hz stimulation for 60 min was most effective. In contrast, Marcolino et al. (2018) reported no significant difference between high- and low-frequency TENS, although their review included more variable protocols and fewer direct comparisons [41]. Zhu et al. (2024) analyzed acupoint-based stimulation in older adults and found that non-invasive electroacupuncture yielded superior spasticity reduction [18]. While not focused on TENS specifically, their findings reinforce the potential of non-invasive sensory-level stimulation. Our study adds value by integrating multiple functional outcomes, analyzing frequency- and time-dependent effects, and including stroke survivors across recovery stages. Unlike earlier reviews, we demonstrate that TENS, particularly at 100 Hz and with adequate session length, can be a broadly effective adjunct for improving spasticity, motor performance.

The robustness of spasticity-related outcomes was confirmed by sensitivity analyses, which demonstrated stable effect estimates regardless of which study was excluded. In contrast, outcomes related to motor function and ADL showed greater sensitivity to individual studies, likely due to the limited number of trials (*n* = 6 and *n* = 4, respectively). Although the funnel plot for spasticity appeared visually symmetrical, Egger’s test indicated significant asymmetry (*P* = 0.008), suggesting the presence of potential publication bias. This discrepancy highlights the limitations of relying solely on visual inspection and underscores the importance of prospective trial registration and transparent reporting practices.

We emphasized that this is one of the few meta-analyses to conduct detailed subgroup analyses based on both stimulation frequency and stroke chronicity. The inclusion of 17 high-quality RCTs and the use of rigorous Cochrane RoB 2.0 methodology further enhance the reliability of our findings. Moreover, the incorporation of sensitivity and publication bias analyses strengthens the robustness of our conclusions. The findings of this review support the incorporation of high-frequency TENS, particularly at 100 Hz, into standard stroke rehabilitation programs, especially as an adjunct to physiotherapy or task-specific training. Given its non-invasive nature, minimal adverse effects, and favorable cost-benefit profile, TENS offers a practical and accessible intervention to enhance neuromotor recovery. Moreover, its demonstrated effectiveness across different phases of stroke recovery suggests a broad therapeutic window for clinical application. Despite these promising results, several gaps in the current evidence base remain. There is a need for future research to explore the dose–response relationship among TENS parameters, including frequency, intensity, and treatment duration. In addition, the relative efficacy of different electrode placements—such as stimulation over peripheral nerves versus acupuncture points—warrants direct comparison in well-controlled trials. Studies with extended follow-up periods are also needed to evaluate the long-term effectiveness and sustainability of TENS-induced functional gains. It is important to acknowledge that the MAS, while widely used in clinical and research settings, has been criticized for its limited ability to measure true spasticity, as it does not differentiate between neural and mechanical components of muscle resistance. This limitation could have influenced the pooled effect estimates, particularly given that our meta-analysis relied on the calculation of SMDs, which assume that all included outcome measures assess the same construct. Although we attempted to mitigate this by including only validated spasticity measures (i.e., MAS and CSS), the inherent differences in what these tools capture may introduce a degree of measurement heterogeneity. Consequently, the interpretation of effect sizes should be made with caution, and future studies are encouraged to incorporate more objective or multi-dimensional assessments, such as the Tardieu Scale or electromyography, to enhance the precision of spasticity measurement.

Future research should explore the potential of combining TENS with other therapeutic modalities to enhance its antispastic effects. While TENS has shown promise in modulating peripheral and spinal excitability, its integration with techniques such as repetitive peripheral magnetic stimulation [42], non-invasive brain stimulation [43–45], or conventional physiotherapy [46, 47] may yield additive or synergistic effects. Furthermore, future trials should adopt comprehensive and multidimensional outcome measures that extend beyond spasticity and motor function to include quality of life, caregiver burden, and neurophysiological markers, in alignment with the International Classification of Functioning, Disability and Health framework. A further limitation is that meta-regression analyses could not be conducted due to the limited number of studies available for certain subgroups. As a result, we were unable to quantitatively explore the influence of study‑level covariates (e.g., stimulation duration, electrode placement, concomitant therapies) on the treatment effect. Methodological rigor must also be prioritized, with careful attention to allocation concealment, blinding of outcome assessors, and adequate sample sizes to ensure sufficient statistical power. We acknowledged the heterogeneity in stimulation protocols (e.g., frequency, intensity, electrode placement), which may affect the generalizability of results. We also discussed the relatively small number of studies assessing motor function and ADL. Another limitation of this meta-analysis concerns the inclusion of two comparisons from the same study population with a shared control group (Yan et al., 2019a and Yan et al., 2019b). Although these substudies investigated different intervention arms, the reuse of the same control data introduces a multiplicity issue that may affect the independence of effect estimates and potentially overstate the precision of pooled results. While we maintained both comparisons in the analysis due to their clinical relevance, we should interpret the results with caution. Despite these limitations, the results of this review have important clinical implications. The collective evidence supports the use of TENS, particularly at high frequencies, as an effective and feasible adjunctive therapy for stroke survivors. Its ease of use and adaptability to both clinical and home-based settings further enhance its potential for broad implementation in neurorehabilitation.

## Conclusions

Our findings reveal that high-frequency stimulation at 100 Hz yields more pronounced improvements in spasticity reduction and motor recovery than lower frequencies. Furthermore, TENS shows significant benefits across all stroke phases, with the most notable effects observed during the acute phase. These results provide clinically relevant evidence supporting the parameter- and phase-specific application of TENS in post-stroke rehabilitation. However, further high-quality studies with standardized protocols are warranted to refine optimal therapeutic parameters and timing.

## Supplementary Information

Below is the link to the electronic supplementary material.

Supplementary Material 1

## Data Availability

The data collected in this study are available from the corresponding author on reasonable request. All primary data were extracted from the referenced sources.

## Abbreviations

TENS: Transcutaneous Electrical Nerve Stimulation
SMD: Standardized Mean Difference
RCT: Randomized Controlled Trial
PEDro: Physiotherapy Evidence Database
ROB: Risk of Bias
MAS: Modified Ashworth Scale
CSS: Composite Spasticity Score

## References

[1] Urban PP, Wolf T, Uebele M, Marx JJ, Vogt T, Stoeter P, et al. Occurence and clinical predictors of spasticity after ischemic stroke. Stroke. 2010;41(9):2016–20. 10.1161/strokeaha.110.581991. Epub 20100812. 20705930 10.1161/STROKEAHA.110.581991

[2] Wissel J, Schelosky LD, Scott J, Christe W, Faiss JH, Mueller J. Early development of spasticity following stroke: a prospective, observational trial. J Neurol. 2010;257(7):1067–72. 10.1007/s00415-010-5463-1. Epub 20100206. 20140444 10.1007/s00415-010-5463-1 PMC2892615

[3] Gillard PJ, Sucharew H, Kleindorfer D, Belagaje S, Varon S, Alwell K, et al. The negative impact of spasticity on the health-related quality of life of stroke survivors: a longitudinal cohort study. Health Qual Life Outcomes. 2015;13:159. 10.1186/s12955-015-0340-3. Epub 20150929. 26415945 10.1186/s12955-015-0340-3 PMC4587810

[4] Mills PB, Dossa F. Transcutaneous electrical nerve stimulation for management of limb spasticity: A systematic review. Am J Phys Med Rehabil. 2016;95(4):309–18. 0000000000000437. PubMed PMID: 26829077. 26829077 10.1097/PHM.0000000000000437

[5] Mahmood A, Veluswamy SK, Hombali A, Mullick A, Solomon NM. Effect of transcutaneous electrical nerve stimulation on spasticity in adults with stroke: A systematic review and Meta-analysis. Arch Phys Med Rehabil. 2019;100(4):751–68. 10.1016/j.apmr.2018.10.016. Epub 20181116. 30452892 10.1016/j.apmr.2018.10.016

[6] Sharififar S, Shuster JJ, Bishop MD. Adding electrical stimulation during standard rehabilitation after stroke to improve motor function. A systematic review and meta-analysis. Ann Phys Rehabil Med. 2018;61(5):339–44. 10.1016/j.rehab.2018.06.005. Epub 20180626. 29958963 10.1016/j.rehab.2018.06.005

[7] Perez MA, Field-Fote EC, Floeter MK. Patterned sensory stimulation induces plasticity in reciprocal ia inhibition in humans. J Neurosci. 2003;23(6):2014-8. 10.1523/jneurosci.23-06-02014.2003. PubMed PMID: 12657659; PubMed Central PMCID: PMCPMC6742007. 10.1523/JNEUROSCI.23-06-02014.2003 PMC6742007 12657659

[8] Crone C, Nielsen J, Petersen N, Ballegaard M, Hultborn H. Disynaptic reciprocal Inhibition of ankle extensors in spastic patients. Brain. 1994;117(Pt 5):1161–8. 10.1093/brain/117.5.1161. PubMed PMID: 7953596. 7953596 10.1093/brain/117.5.1161

[9] Mima T, Oga T, Rothwell J, Satow T, Yamamoto J, Toma K, et al. Short-term high-frequency transcutaneous electrical nerve stimulation decreases human motor cortex excitability. Neurosci Lett. 2004;355(1–2):85–8. 10.1016/j.neulet.2003.10.045. PubMed PMID: 14729241. 14729241 10.1016/j.neulet.2003.10.045

[10] Tinazzi M, Zarattini S, Valeriani M, Romito S, Farina S, Moretto G et al. Long-lasting modulation of human motor cortex following prolonged transcutaneous electrical nerve stimulation (TENS) of forearm muscles: evidence of reciprocal inhibition and facilitation. Exp Brain Res. 2005;161(4):457 – 64. Epub 20041116. 10.1007/s00221-004-2091-y. PubMed PMID: 15551083. 10.1007/s00221-004-2091-y 15551083

[11] Golaszewski S, Kremser C, Wagner M, Felber S, Aichner F, Dimitrijevic MM. Functional magnetic resonance imaging of the human motor cortex before and after whole-hand afferent electrical stimulation. Scand J Rehabil Med. 1999;31(3):165–73. doi: 10.1080/003655099444506. PubMed PMID: 10458314. 10458314 10.1080/003655099444506

[12] Koyama S, Tanabe S, Takeda K, Sakurai H, Kanada Y. Modulation of spinal inhibitory reflexes depends on the frequency of transcutaneous electrical nerve stimulation in spastic stroke survivors. Somatosens Mot Res. 2016;33(1):8–15. 10.3109/08990220.2016.1142436. Epub 20160307. 26949041 10.3109/08990220.2016.1142436

[13] Jung KS, Jung JH, Cho HY, In TS. Effects of transcutaneous electrical nerve stimulation with taping on wrist spasticity, strength, and upper extremity function in patients with stroke: A randomized control trial. J Clin Med. 2024;13(8). 10.3390/jcm13082229. Epub 20240412. 10.3390/jcm13082229 PMC11051346 38673502

[14] Kwong PW, Ng GY, Chung RC, Ng SS. Transcutaneous electrical nerve stimulation improves walking capacity and reduces spasticity in stroke survivors: a systematic review and meta-analysis. Clin Rehabil. 2018;32(9):1203–19. Epub 20171213. doi: 10.1177/0269215517745349. PubMed PMID: 29232981. 29232981 10.1177/0269215517745349

[15] Marcolino MAZ, Hauck M, Stein C, Schardong J, Pagnussat AS, Plentz RDM. Effects of transcutaneous electrical nerve stimulation alone or as additional therapy on chronic post-stroke spasticity: systematic review and meta-analysis of randomized controlled trials. Disabil Rehabil. 2020;42(5):623–35. 1503736. PubMed PMID: 30326752. 30326752 10.1080/09638288.2018.1503736

[16] Roman N, Miclaus RS, Necula R, Dumistracel A, Cheregi C, Grigorescu OD. Physiotherapy efficiency in Post-stroke upper extremity spasticity: TENS vs. Ultrasound vs. Paraffin. Vivo. 2023;37(2):916–23. 10.21873/invivo.13163. PubMed PMID: 36881086; PubMed Central PMCID: PMCPMC10026645. 10.21873/invivo.13163 PMC10026645 36881086

[17] Senarath ID, Thalwathte RD, Pathirage M, Kularatne SAM. The effectiveness of radial extracorporeal shock wave therapy vs transcutaneous electrical nerve stimulation in the management of upper limb spasticity in chronic-post stroke hemiplegia-A randomized controlled trial. PLoS ONE. 2023;18(5):e0283321. 10.1371/journal.pone.0283321. Epub 20230526. 37235581 10.1371/journal.pone.0283321 PMC10218748

[18] Zhu GC, Chen KM, Belcastro F. Comparing the effects of different acupoint-stimulating therapies in mitigating post-stroke spasticity and motor dysfunction in older stroke survivors: A network meta-analysis of randomized trials. Maturitas. 2024;187:108040. 10.1016/j.maturitas.2024.108040. Epub 20240605. 38852490 10.1016/j.maturitas.2024.108040

[19] Laufer Y, Elboim-Gabyzon M. Does sensory transcutaneous electrical stimulation enhance motor recovery following a stroke? A systematic review. Neurorehabil Neural Repair. 2011;25(9):799–809. Epub 20110711. doi: 10.1177/1545968310397205. PubMed PMID: 21746874. 21746874 10.1177/1545968310397205

[20] Mahmood A, Veluswamy SK, Hombali A, Mullick A, Solomon NM. Effect of transcutaneous electrical nerve stimulation on spasticity in adults with stroke: A systematic review and Meta-analysis. Arch Phys Med Rehabil. 2019;100(4):751–68. 10.1016/j.apmr.2018.10.016. 30452892 10.1016/j.apmr.2018.10.016

[21] Cumpston M, Li T, Page MJ, Chandler J, Welch VA, Higgins JP, et al. Updated guidance for trusted systematic reviews: a new edition of the Cochrane handbook for systematic reviews of interventions. Cochrane Database Syst Rev. 2019;10(10):Ed000142. PubMed PMID: 31643080; PubMed Central PMCID: PMCPMC10284251. 31643080 10.1002/14651858.ED000142 PMC10284251

[22] Page MJ, McKenzie JE, Bossuyt PM, Boutron I, Hoffmann TC, Mulrow CD, et al. The PRISMA 2020 statement: an updated guideline for reporting systematic reviews. BMJ. 2021;372. 10.1136/bmj.n71. Epub 20210329. :n71. 10.1136/bmj.n71 PMC8005924 33782057

[23] Baricich A, Carda S, Bertoni M, Maderna L, Cisari C. A single-blinded, randomized pilot study of botulinum toxin type A combined with non-pharmacological treatment for spastic foot. J Rehabil Med. 2008;40(10):870–2. 10.2340/16501977-0251. PubMed PMID: 19242626. 19242626 10.2340/16501977-0251

[24] Chen SC, Chen YL, Chen CJ, Lai CH, Chiang WH, Chen WL. Effects of surface electrical stimulation on the muscle-tendon junction of spastic gastrocnemius in stroke patients. Disabil Rehabil. 2005;27(3):105–10. 10.1080/09638280400009022. PubMed PMID: 15823991. 15823991 10.1080/09638280400009022

[25] Cho H, In TS, Cho KH, Song CH. A single trial of transcutaneous electrical nerve stimulation (TENS) improves spasticity and balance in patients with chronic stroke. Tohoku J Exp Med. 2013;229(3):187–93. 10.1620/tjem.229.187. 23419328 10.1620/tjem.229.187

[26] de Jong LD, Dijkstra PU, Gerritsen J, Geurts AC, Postema K. Combined arm stretch positioning and neuromuscular electrical stimulation during rehabilitation does not improve range of motion, shoulder pain or function in patients after stroke: a randomised trial. J Physiother. 2013;59(4):245–54. 10.1016/s1836-9553(13)70201-7. PubMed PMID: 24287218. 24287218 10.1016/S1836-9553(13)70201-7

[27] Deshmukh MK, Kumar C, Goyal M, editors APPLICATION OF TRANSCUTANEOUS ELECTRICAL STIMULATION ON LOWER LIMB ACUPOINTS AS AN IMPORTANT ADJUNCTIVE TOOL IN STROKE REHABILITATION PROGRAM. & ITS EFFECTS ON SPASTICITY AND FUNCTIONAL ABILITY2013.

[28] Hui-Chan CW, Ng SS, Mak MK. Effectiveness of a home-based rehabilitation programme on lower limb functions after stroke. Hong Kong Med J. 2009;15(3 Suppl 4):42–6. PubMed PMID: CN-01159453. 19509438

[29] Hussain T, Mohammad H. The effect of transcutaneous electrical nerve stimulation (TENS) combined with Bobath on post stroke spasticity. A randomized controlled study. J Neurol Sci. 2013;333:e560. PubMed PMID: CN-01006692.

[30] Jung KS, In TS, Cho HY. Effects of sit-to-stand training combined with transcutaneous electrical stimulation on spasticity, muscle strength and balance ability in patients with stroke: A randomized controlled study. Gait Posture. 2017;54:183–7. 10.1016/j.gaitpost.2017.03.007. Epub 20170310. 28324754 10.1016/j.gaitpost.2017.03.007

[31] Kim TH, In TS, Cho HY. Task-related training combined with transcutaneous electrical nerve stimulation promotes upper limb functions in patients with chronic stroke. Tohoku J Exp Med. 2013;231(2):93–100. 10.1620/tjem.231.93. PubMed PMID: 24097280. 24097280 10.1620/tjem.231.93

[32] Ng SSM, Hui-Chan CWY. Transcutaneous electrical nerve stimulation combined with Task-Related training improves lower limb functions in subjects with chronic stroke. Stroke. 2007;38(11):2953–9. 10.1161/STROKEAHA.107.490318. 17901383 10.1161/STROKEAHA.107.490318

[33] Park J, Seo D, Choi W, Lee S. The effects of exercise with TENS on spasticity, balance, and gait in patients with chronic stroke: A randomized controlled trial. Med Sci Monit. 2014;20. PubMed PMID: WOS:000344494800001. 10.12659/MSM.890926 PMC4206395 25300431

[34] Sonde L, Kalimo H, Fernaeus SE, Viitanen M. Low TENS treatment on post-stroke Paretic arm: a three-year follow-up. Clin Rehabil. 2000;14(1):14–9. doi: 10.1191/026921500673534278. PubMed PMID: 10688340. 10688340 10.1191/026921500673534278

[35] Tekeoòlu Ý, Adak B, Göksoy T. Effect of transcutaneous electrical nerve stimulation (TENS) on Barthel activities of daily living (ADL) index score following stroke. Clin Rehabil. 1998;12(4):277–80. 10.1191/026921598672873816. 9744663 10.1191/026921598672873816

[36] Wang H, Xiang Y, Wang C, Wang Y, Chen S, Ding L, et al. Effects of transcutaneous electrical acupoint stimulation on upper-limb impairment after stroke: A randomized, controlled, single-blind trial. Clin Rehabil. 2023;37(5):667–78. 10.1177/02692155221138916. PubMed PMID: 36380681. 36380681 10.1177/02692155221138916 PMC10041575

[37] Wang Y, Zhang L, Yin R, Zhang Y, Dai Z, Wang M, et al. Transcutaneous electrical acupoint stimulation for upper limb spasticity after stroke: effect and feasibility-a randomised pilot study. BMJ Support Palliat Care. 2025;15(2):237–44. 10.1136/spcare-2024-005174. Epub 20250226. 39798944 10.1136/spcare-2024-005174 PMC11874307

[38] Yan T, Hui-Chan CWY. Transcutaneous electrical stimulation on acupuncture points improves muscle function in subjects after acute stroke: A randomized controlled trial. J Rehabil Med. 2009;41(5):312–6. 10.2340/16501977-0325. 19363561 10.2340/16501977-0325

[39] Zhou M, Li F, Lu W, Wu J, Pei S. Efficiency of neuromuscular electrical stimulation and transcutaneous nerve stimulation on hemiplegic shoulder pain: A randomized controlled trial. Arch Phys Med Rehabil. 2018;99(9):1730–9. 10.1016/j.apmr.2018.04.020. 29777714 10.1016/j.apmr.2018.04.020

[40] Kwong PWH, Ng GYF, Chung RCK, Ng SSM. Transcutaneous electrical nerve stimulation improves walking capacity and reduces spasticity in stroke survivors: a systematic review and meta-analysis. Clin Rehabil. 2018;32(9):1203–19. 10.1177/0269215517745349. 29232981 10.1177/0269215517745349

[41] Marcolino MAZ, Hauck M, Stein C, Schardong J, Pagnussat AS, Plentz RDM. Effects of transcutaneous electrical nerve stimulation alone or as additional therapy on chronic post-stroke spasticity: systematic review and meta-analysis of randomized controlled trials. Disabil Rehabil. 2020;42(5):623–35. 10.1080/09638288.2018.1503736. 30326752 10.1080/09638288.2018.1503736

[42] Pan J-X, Diao Y-X, Peng H-Y, Wang X-Z, Liao L-R, Wang M-Y, et al. Effects of repetitive peripheral magnetic stimulation on spasticity evaluated with modified Ashworth scale/ashworth scale in patients with spastic paralysis: A systematic review and meta-analysis. Front Neurol. 2022;13–2022. 10.3389/fneur.2022.997913. 10.3389/fneur.2022.997913 PMC9679494 36425797

[43] Chen YJ, Huang YZ, Chen CY, Chen CL, Chen HC, Wu CY, et al. Intermittent theta burst stimulation enhances upper limb motor function in patients with chronic stroke: a pilot randomized controlled trial. BMC Neurol. 2019;19(1):69. 10.1186/s12883-019-1302-x. Epub 20190425. 31023258 10.1186/s12883-019-1302-x PMC6485156

[44] Barros Galvão SC, Borba Costa dos Santos R, Borba dos Santos P, Cabral ME, Monte-Silva K. Efficacy of coupling repetitive transcranial magnetic stimulation and physical therapy to reduce upper-limb spasticity in patients with stroke: a randomized controlled trial. Arch Phys Med Rehabil. 2014;95(2):222–9. 10.1016/j.apmr.2013.10.023. Epub 20131112. 24239881 10.1016/j.apmr.2013.10.023

[45] Elsner B, Kugler J, Pohl M, Mehrholz J. Transcranial direct current stimulation for improving spasticity after stroke: A systematic review with meta-analysis. J Rehabil Med. 2016;48(7):565–70. PubMed PMID: 27172484. 27172484 10.2340/16501977-2097

[46] Chen J, Or CK, Chen T. Effectiveness of using virtual Reality-Supported exercise therapy for upper extremity motor rehabilitation in patients with stroke: systematic review and Meta-analysis of randomized controlled trials. J Med Internet Res. 2022;24(6):e24111. Epub 20220620. doi: 10.2196/24111. PubMed PMID: 35723907; PubMed Central PMCID: PMCPMC9253973. 35723907 10.2196/24111 PMC9253973

[47] Salazar AP, Pinto C, Ruschel Mossi JV, Figueiro B, Lukrafka JL, Pagnussat AS. Effectiveness of static stretching positioning on post-stroke upper-limb spasticity and mobility: systematic review with meta-analysis. Ann Phys Rehabil Med. 2019;62(4):274–82. PubMed PMID: 30582986. 30582986 10.1016/j.rehab.2018.11.004

