# Supplementary material for: Superior efficacy of 100-Hz transcutaneous electrical nerve stimulation in reducing post-stroke spasticity: a systematic review and meta-analysis
Source: J Neuroeng Rehabil. 2025 Oct 9;22:210. doi: 10.1186/s12984-025-01744-3 (PMC12512259; doi:10.1186/s12984-025-01744-3)
Supplement: Supplementary file 1 — Supplementary Material 1 [file 12984_2025_1744_MOESM1_ESM.docx]

**Search Strategy**

**Pubmed**

("Stroke"[MeSH Terms] OR "Strokes"[All Fields] OR "Cerebrovascular Accident"[All Fields] OR "Cerebrovascular Accidents"[All Fields] OR "Cerebral Stroke"[All Fields] OR "Cerebral Strokes"[All Fields] OR "stroke cerebral"[All Fields] OR "strokes cerebral"[All Fields] OR "Cerebrovascular Apoplexy"[All Fields] OR "apoplexy cerebrovascular"[All Fields] OR "vascular accident brain"[All Fields] OR "Brain Vascular Accident"[All Fields] OR "Brain Vascular Accidents"[All Fields] OR ("Stroke"[MeSH Terms] OR "Stroke"[All Fields] OR ("vascular"[All Fields] AND "accidents"[All Fields] AND "brain"[All Fields])) OR "Cerebrovascular Stroke"[All Fields] OR "Cerebrovascular Strokes"[All Fields] OR "stroke cerebrovascular"[All Fields] OR "strokes cerebrovascular"[All Fields] OR "Apoplexy"[All Fields] OR "cva cerebrovascular accident"[All Fields] OR ("Stroke"[MeSH Terms] OR "Stroke"[All Fields] OR ("cvas"[All Fields] AND "cerebrovascular"[All Fields] AND "accident"[All Fields])) OR "stroke acute"[All Fields] OR "Acute Stroke"[All Fields] OR "Acute Strokes"[All Fields] OR "strokes acute"[All Fields] OR "cerebrovascular accident acute"[All Fields] OR "Acute Cerebrovascular Accident"[All Fields] OR "Acute Cerebrovascular Accidents"[All Fields] OR "cerebrovascular accidents acute"[All Fields]) AND ("Transcutaneous Electric Nerve Stimulation"[MeSH Terms] OR "Transcutaneous Nerve Stimulation"[All Fields] OR "nerve stimulation transcutaneous"[All Fields] OR "stimulation transcutaneous nerve"[All Fields] OR "electric stimulation transcutaneous"[All Fields] OR "stimulation transcutaneous electric"[All Fields] OR "Transcutaneous Electric Stimulation"[All Fields] OR "Percutaneous Electric Nerve Stimulation"[All Fields] OR "TENS"[All Fields] OR "Transdermal Electrostimulation"[All Fields] OR ("Transcutaneous Electric Nerve Stimulation"[MeSH Terms] OR ("transcutaneous"[All Fields] AND "electric"[All Fields] AND "nerve"[All Fields] AND "stimulation"[All Fields]) OR "Transcutaneous Electric Nerve Stimulation"[All Fields] OR ("electrostimulation"[All Fields] AND "transdermal"[All Fields])) OR "Percutaneous Electrical Nerve Stimulation"[All Fields] OR "Transcutaneous Electrical Nerve Stimulation"[All Fields] OR "electrical stimulation transcutaneous"[All Fields] OR "Transcutaneous Electrical Stimulation"[All Fields] OR "Analgesic Cutaneous Electrostimulation"[All Fields] OR ("Transcutaneous Electric Nerve Stimulation"[MeSH Terms] OR ("transcutaneous"[All Fields] AND "electric"[All Fields] AND "nerve"[All Fields] AND "stimulation"[All Fields]) OR "Transcutaneous Electric Nerve Stimulation"[All Fields] OR ("cutaneous"[All Fields] AND "electrostimulation"[All Fields] AND "analgesic"[All Fields])) OR ("Transcutaneous Electric Nerve Stimulation"[MeSH Terms] OR ("transcutaneous"[All Fields] AND "electric"[All Fields] AND "nerve"[All Fields] AND "stimulation"[All Fields]) OR "Transcutaneous Electric Nerve Stimulation"[All Fields] OR ("electrostimulation"[All Fields] AND "analgesic"[All Fields] AND "cutaneous"[All Fields])) OR "Electroanalgesia"[All Fields] OR ("Transcutaneous Electric Nerve Stimulation"[MeSH Terms] OR ("transcutaneous"[All Fields] AND "electric"[All Fields] AND "nerve"[All Fields] AND "stimulation"[All Fields]) OR "Transcutaneous Electric Nerve Stimulation"[All Fields]) OR "Percutaneous Neuromodulation Therapy"[All Fields] OR ("Transcutaneous Electric Nerve Stimulation"[MeSH Terms] OR ("transcutaneous"[All Fields] AND "electric"[All Fields] AND "nerve"[All Fields] AND "stimulation"[All Fields]) OR "Transcutaneous Electric Nerve Stimulation"[All Fields] OR ("neuromodulation"[All Fields] AND "therapy"[All Fields] AND "percutaneous"[All Fields])) OR "Percutaneous Neuromodulation Therapies"[All Fields] OR ("Transcutaneous Electric Nerve Stimulation"[MeSH Terms] OR ("transcutaneous"[All Fields] AND "electric"[All Fields] AND "nerve"[All Fields] AND "stimulation"[All Fields]) OR "Transcutaneous Electric Nerve Stimulation"[All Fields] OR ("therapy"[All Fields] AND "percutaneous"[All Fields] AND "neuromodulation"[All Fields])) OR "Percutaneous Electrical Neuromodulation"[All Fields] OR ("Transcutaneous Electric Nerve Stimulation"[MeSH Terms] OR ("transcutaneous"[All Fields] AND "electric"[All Fields] AND "nerve"[All Fields] AND "stimulation"[All Fields]) OR "Transcutaneous Electric Nerve Stimulation"[All Fields] OR ("electrical"[All Fields] AND "neuromodulation"[All Fields] AND "percutaneous"[All Fields])) OR ("Transcutaneous Electric Nerve Stimulation"[MeSH Terms] OR ("transcutaneous"[All Fields] AND "electric"[All Fields] AND "nerve"[All Fields] AND "stimulation"[All Fields]) OR "Transcutaneous Electric Nerve Stimulation"[All Fields] OR ("electrical"[All Fields] AND "neuromodulations"[All Fields] AND "percutaneous"[All Fields])) OR ("Transcutaneous Electric Nerve Stimulation"[MeSH Terms] OR ("transcutaneous"[All Fields] AND "electric"[All Fields] AND "nerve"[All Fields] AND "stimulation"[All Fields]) OR "Transcutaneous Electric Nerve Stimulation"[All Fields] OR ("neuromodulation"[All Fields] AND "percutaneous"[All Fields] AND "electrical"[All Fields])) OR ("Transcutaneous Electric Nerve Stimulation"[MeSH Terms] OR ("transcutaneous"[All Fields] AND "electric"[All Fields] AND "nerve"[All Fields] AND "stimulation"[All Fields]) OR "Transcutaneous Electric Nerve Stimulation"[All Fields] OR ("neuromodulations"[All Fields] AND "percutaneous"[All Fields] AND "electrical"[All Fields])) OR ("Transcutaneous Electric Nerve Stimulation"[MeSH Terms] OR ("transcutaneous"[All Fields] AND "electric"[All Fields] AND "nerve"[All Fields] AND "stimulation"[All Fields]) OR "Transcutaneous Electric Nerve Stimulation"[All Fields] OR ("percutaneous"[All Fields] AND "electrical"[All Fields] AND "neuromodulations"[All Fields])))

**Cochrane Library**

#1 Stroke

#2 Transcutaneous Electrical Nerve Stimulation

#3 #1 AND #2

**PEDro**

[Transcutaneous electric nerve stimulation](https://search.pedro.org.au/search-results/record-detail/20856)

**Web of Science**

TS=("Stroke" OR "Cerebrovascular Accident" OR "Cerebrovascular Accidents" OR "Cerebral Stroke" OR "Cerebral Strokes" OR "Stroke Cerebral" OR "Strokes Cerebral" OR "Cerebrovascular Apoplexy" OR "Apoplexy Cerebrovascular" OR "Vascular Accident Brain" OR "Brain Vascular Accident" OR "Brain Vascular Accidents" OR "Cerebrovascular Stroke" OR "Cerebrovascular Strokes" OR "Stroke Cerebrovascular" OR "Strokes Cerebrovascular" OR "Apoplexy" OR "CVA" OR "Acute Stroke" OR "Acute Strokes" OR "Strokes Acute" OR "Cerebrovascular Accident Acute" OR "Acute Cerebrovascular Accident" OR "Acute Cerebrovascular Accidents" OR "Cerebrovascular Accidents Acute")

TS=("Transcutaneous Electric Nerve Stimulation" OR "TENS" OR "Transcutaneous Nerve Stimulation" OR "Nerve Stimulation Transcutaneous" OR "Stimulation Transcutaneous Nerve" OR "Electric Stimulation Transcutaneous" OR "Transcutaneous Electric Stimulation" OR "Percutaneous Electric Nerve Stimulation" OR "Transdermal Electrostimulation" OR "Percutaneous Electrical Nerve Stimulation" OR "Transcutaneous Electrical Nerve Stimulation" OR "Electrical Stimulation Transcutaneous" OR "Transcutaneous Electrical Stimulation" OR "Analgesic Cutaneous Electrostimulation" OR "Electroanalgesia" OR "Percutaneous Neuromodulation Therapy" OR "Percutaneous Electrical Neuromodulation")

**Embase**

('stroke'/exp OR 'cerebrovascular accident':ti,ab OR 'cerebrovascular accidents':ti,ab OR

'cerebral stroke':ti,ab OR 'cerebral strokes':ti,ab OR 'stroke cerebral':ti,ab OR 'strokes cerebral':ti,ab OR 'cerebrovascular apoplexy':ti,ab OR 'apoplexy cerebrovascular':ti,ab OR 'vascular accident brain':ti,ab OR 'brain vascular accident':ti,ab OR 'brain vascular accidents':ti,ab OR 'cerebrovascular stroke':ti,ab OR 'cerebrovascular strokes':ti,ab OR 'stroke cerebrovascular':ti,ab OR 'strokes cerebrovascular':ti,ab OR 'apoplexy':ti,ab OR 'cva cerebrovascular accident':ti,ab OR 'acute stroke':ti,ab OR 'acute strokes':ti,ab OR 'strokes acute':ti,ab OR 'cerebrovascular accident acute':ti,ab OR 'acute cerebrovascular accident':ti,ab OR 'acute cerebrovascular accidents':ti,ab OR 'cerebrovascular accidents acute':ti,ab)

AND

('transcutaneous electric nerve stimulation'/exp OR 'tens':ti,ab OR 'transcutaneous nerve stimulation':ti,ab OR 'nerve stimulation transcutaneous':ti,ab OR 'stimulation transcutaneous nerve':ti,ab OR 'electric stimulation transcutaneous':ti,ab OR 'stimulation transcutaneous electric':ti,ab OR 'transcutaneous electric stimulation':ti,ab OR 'percutaneous electric nerve stimulation':ti,ab OR 'transdermal electrostimulation':ti,ab OR 'percutaneous electrical nerve stimulation':ti,ab OR 'transcutaneous electrical nerve stimulation':ti,ab OR 'electrical stimulation transcutaneous':ti,ab OR 'transcutaneous electrical stimulation':ti,ab OR 'analgesic cutaneous electrostimulation':ti,ab OR 'electroanalgesia':ti,ab OR 'percutaneous neuromodulation therapy':ti,ab OR 'percutaneous electrical neuromodulation':ti,ab)

**Scopus**

TITLE-ABS-KEY ("Stroke" OR "Cerebrovascular Accident" OR "Cerebrovascular Accidents" OR "Cerebral Stroke" OR "Cerebral Strokes" OR "Stroke Cerebral" OR "Strokes Cerebral" OR "Cerebrovascular Apoplexy" OR "Apoplexy Cerebrovascular" OR "Vascular Accident Brain" OR "Brain Vascular Accident" OR "Brain Vascular Accidents" OR "Cerebrovascular Stroke" OR "Cerebrovascular Strokes" OR "Stroke Cerebrovascular" OR "Strokes Cerebrovascular" OR "Apoplexy" OR "CVA" OR "Acute Stroke" OR "Acute Strokes" OR "Strokes Acute" OR "Cerebrovascular Accident Acute" OR "Acute Cerebrovascular Accident" OR "Acute Cerebrovascular Accidents" OR "Cerebrovascular Accidents Acute") AND TITLE-ABS-KEY ("Transcutaneous Electric Nerve Stimulation" OR "TENS" OR "Transcutaneous Nerve Stimulation" OR "Nerve Stimulation Transcutaneous" OR "Stimulation Transcutaneous Nerve" OR "Electric Stimulation Transcutaneous" OR "Transcutaneous Electric Stimulation" OR "Percutaneous Electric Nerve Stimulation" OR "Transdermal Electrostimulation" OR "Percutaneous Electrical Nerve Stimulation" OR "Transcutaneous Electrical Nerve Stimulation" OR "Electrical Stimulation Transcutaneous" OR "Transcutaneous Electrical Stimulation" OR "Analgesic Cutaneous Electrostimulation" OR "Electroanalgesia" OR "Percutaneous Neuromodulation Therapy" OR "Percutaneous Electrical Neuromodulation")

**Table S2 List of excluded articles with reasons**

| **#** | **Author(s)** | **Article title** | **Reason for exclusion** |
| --- | --- | --- | --- |
| 1 | Kwong et al., 2022 | Immediate effects of transcutaneous electrical nerve stimulation  on gait patterns in chronic stroke survivors: A single group,  pretest-posttest clinical trial | No control group; single-group pretest-posttest design |
| 2 | In et al., 2021 | Effectiveness of Transcutaneous Electrical Nerve Stimulation with Taping for Stroke Rehabilitation | The comparison was TENS + taping vs. TENS alone; both groups received TENS, violating the requirement for a true non-TENS control group |
| 3 | Ersoy et al., 2023 | Comparison of transcutaneous electrical stimulation and suprascapular nerve blockage for the treatment of hemiplegic shoulder pain | Control group received suprascapular nerve block—not a true placebo; outcome data on spasticity were reported as percentages, incompatible with pooled continuous data analysis |
| 4 | ErtZGaard et al., 2018 | Evaluation of a self-administered transcutaneous electrical stimulation concept for the treatment of spasticity: a randomized placebo-controlled trial | Sequential design where both groups received TENS; not a valid control; excluded due to design inconsistency |
| 5 | Senarath et al., 2023 | The effectiveness of radial extracorporeal shock wave therapy vs transcutaneous electrical nerve stimulation in the management of upper limb spasticity in chronic-post stroke hemiplegia–A randomized controlled trial | TENS was used as the control group; not suitable for inclusion |
| 6 | Johansson et al., 2001 | Acupuncture and Transcutaneous Nerve Stimulation in Stroke Rehabilitation | No extractable data on spasticity outcomes |
| 7 | Marco et al., 2007 | Is botulinum toxin type a effective in the treatment of spastic shoulder pain in patients after stroke? a double-blind randomized clinical trial | Both intervention and control groups received TENS (BTA + TENS vs. Placebo + TENS); this design violates the inclusion criteria, which require the control group to be free of TENS. |
| 8 | Xia et al., 2023 | Clinical study of low-frequency acupoint electrical stimulation to improve thumb-to-finger movements after stroke: A randomized controlled trial | Spasticity data were ordinal and could not be included in continuous data meta-analysis |
| 9 | Laddha et al., 2015 | Effect of Transcutaneous Electrical Nerve Stimulation on  Plantar Flexor Muscle Spasticity and Walking Speed in  Stroke Patients | Spasticity outcome was reported only as a figure; numerical data were not extractable |
| 10 | Picelli et al., 2016 | Efficacy of Therapeutic Ultrasound and Transcutaneous Electrical Nerve Stimulation Compared With Botulinum  Toxin Type A in the Treatment of Spastic Equinus in Adults With Chronic Stroke: A Pilot Randomized Controlled Trial | Outcome data could not be extracted from the publication |

| **Section and Topic** | **Item #** | **Checklist item** | **Location where item is reported** |
| --- | --- | --- | --- |
| **TITLE** | | |  |
| Title | 1 | Identify the report as a systematic review. | 1 |
| **ABSTRACT** | | |  |
| Abstract | 2 | See the PRISMA 2020 for Abstracts checklist. | 2 |
| **INTRODUCTION** | | |  |
| Rationale | 3 | Describe the rationale for the review in the context of existing knowledge. | 3 |
| Objectives | 4 | Provide an explicit statement of the objective(s) or question(s) the review addresses. | 4 |
| **METHODS** | | |  |
| Eligibility criteria | 5 | Specify the inclusion and exclusion criteria for the review and how studies were grouped for the syntheses. | 4 |
| Information sources | 6 | Specify all databases, registers, websites, organisations, reference lists and other sources searched or consulted to identify studies. Specify the date when each source was last searched or consulted. | 4 |
| Search strategy | 7 | Present the full search strategies for all databases, registers and websites, including any filters and limits used. | 5 |
| Selection process | 8 | Specify the methods used to decide whether a study met the inclusion criteria of the review, including how many reviewers screened each record and each report retrieved, whether they worked independently, and if applicable, details of automation tools used in the process. | 5 |
| Data collection process | 9 | Specify the methods used to collect data from reports, including how many reviewers collected data from each report, whether they worked independently, any processes for obtaining or confirming data from study investigators, and if applicable, details of automation tools used in the process. | 5 |
| Data items | 10a | List and define all outcomes for which data were sought. Specify whether all results that were compatible with each outcome domain in each study were sought (e.g. for all measures, time points, analyses), and if not, the methods used to decide which results to collect. | 6 |
|  | 10b | List and define all other variables for which data were sought (e.g. participant and intervention characteristics, funding sources). Describe any assumptions made about any missing or unclear information. | 6 |
| Study risk of bias assessment | 11 | Specify the methods used to assess risk of bias in the included studies, including details of the tool(s) used, how many reviewers assessed each study and whether they worked independently, and if applicable, details of automation tools used in the process. | 6 |
| Effect measures | 12 | Specify for each outcome the effect measure(s) (e.g. risk ratio, mean difference) used in the synthesis or presentation of results. | 6 |
| Synthesis methods | 13a | Describe the processes used to decide which studies were eligible for each synthesis (e.g. tabulating the study intervention characteristics and comparing against the planned groups for each synthesis (item #5)). | 6 |
|  | 13b | Describe any methods required to prepare the data for presentation or synthesis, such as handling of missing summary statistics, or data conversions. | 6 |
|  | 13c | Describe any methods used to tabulate or visually display results of individual studies and syntheses. | 6 |
|  | 13d | Describe any methods used to synthesize results and provide a rationale for the choice(s). If meta-analysis was performed, describe the model(s), method(s) to identify the presence and extent of statistical heterogeneity, and software package(s) used. | 6 |
|  | 13e | Describe any methods used to explore possible causes of heterogeneity among study results (e.g. subgroup analysis, meta-regression). | 6 |
|  | 13f | Describe any sensitivity analyses conducted to assess robustness of the synthesized results. | 6 |
| Reporting bias assessment | 14 | Describe any methods used to assess risk of bias due to missing results in a synthesis (arising from reporting biases). | 6 |
| Certainty assessment | 15 | Describe any methods used to assess certainty (or confidence) in the body of evidence for an outcome. | 6 |
| **RESULTS** | | |  |
| Study selection | 16a | Describe the results of the search and selection process, from the number of records identified in the search to the number of studies included in the review, ideally using a flow diagram. | 7 |
|  | 16b | Cite studies that might appear to meet the inclusion criteria, but which were excluded, and explain why they were excluded. | 7 |
| Study characteristics | 17 | Cite each included study and present its characteristics. | 8 |
| Risk of bias in studies | 18 | Present assessments of risk of bias for each included study. | 8 |
| Results of individual studies | 19 | For all outcomes, present, for each study: (a) summary statistics for each group (where appropriate) and (b) an effect estimate and its precision (e.g. confidence/credible interval), ideally using structured tables or plots. | 8 |
| Results of syntheses | 20a | For each synthesis, briefly summarise the characteristics and risk of bias among contributing studies. | 8 |
|  | 20b | Present results of all statistical syntheses conducted. If meta-analysis was done, present for each the summary estimate and its precision (e.g. confidence/credible interval) and measures of statistical heterogeneity. If comparing groups, describe the direction of the effect. | 9 |
|  | 20c | Present results of all investigations of possible causes of heterogeneity among study results. | 9 |
|  | 20d | Present results of all sensitivity analyses conducted to assess the robustness of the synthesized results. | 10 |
| Reporting biases | 21 | Present assessments of risk of bias due to missing results (arising from reporting biases) for each synthesis assessed. | 10 |
| Certainty of evidence | 22 | Present assessments of certainty (or confidence) in the body of evidence for each outcome assessed. | 10 |
| **DISCUSSION** | | |  |
| Discussion | 23a | Provide a general interpretation of the results in the context of other evidence. | 11 |
|  | 23b | Discuss any limitations of the evidence included in the review. | 11 |
|  | 23c | Discuss any limitations of the review processes used. | 12 |
|  | 23d | Discuss implications of the results for practice, policy, and future research. | 12 |
| **OTHER INFORMATION** | | |  |
| Registration and protocol | 24a | Provide registration information for the review, including register name and registration number, or state that the review was not registered. | 1 |
|  | 24b | Indicate where the review protocol can be accessed, or state that a protocol was not prepared. | 1 |
|  | 24c | Describe and explain any amendments to information provided at registration or in the protocol. | 1 |
| Support | 25 | Describe sources of financial or non-financial support for the review, and the role of the funders or sponsors in the review. | 13 |
| Competing interests | 26 | Declare any competing interests of review authors. | Cover Letter |
| Availability of data, code and other materials | 27 | Report which of the following are publicly available and where they can be found: template data collection forms; data extracted from included studies; data used for all analyses; analytic code; any other materials used in the review. | 13 |

*From:*  Page MJ, McKenzie JE, Bossuyt PM, Boutron I, Hoffmann TC, Mulrow CD, et al. The PRISMA 2020 statement: an updated guideline for reporting systematic reviews. BMJ 2021;372:n71. doi: 10.1136/bmj.n71. This work is licensed under CC BY 4.0. To view a copy of this license, visit <https://creativecommons.org/licenses/by/4.0/>
